# Partial Boolean Functions With Exact Quantum Query Complexity One

**DOI:** 10.3390/e23020189

**Published:** 2021-02-03

**Authors:** Guoliang Xu, Daowen Qiu

**Affiliations:** 1 Institute of Quantum Computing and Computer Theory, School of Computer Science and Engineering, Sun Yat-sen University, Guangzhou 510006, China; xu1guo2liang@foxmail.com; 2 Guangdong Key Laboratory of Information Security Technology, Sun Yat-sen University, Guangzhou 510006, China

**Keywords:** quantum computation, quantum query complexity, quantum query algorithm, partial Boolean function

## Abstract

We provide two sufficient and necessary conditions to characterize any *n*-bit partial Boolean function with exact quantum query complexity 1. Using the first characterization, we present all *n*-bit partial Boolean functions that depend on *n* bits and can be computed exactly by a 1-query quantum algorithm. Due to the second characterization, we construct a function *F* that maps any *n*-bit partial Boolean function to some integer, and if an *n*-bit partial Boolean function *f* depends on *k* bits and can be computed exactly by a 1-query quantum algorithm, then F(f) is non-positive. In addition, we show that the number of all *n*-bit partial Boolean functions that depend on *k* bits and can be computed exactly by a 1-query quantum algorithm is not bigger than an upper bound depending on *n* and *k*. Most importantly, the upper bound is far less than the number of all *n*-bit partial Boolean functions for all efficiently big *n*.

## 1. Introduction

In the field of theoretical computer science, computational complexity aims to measure “how much" computation is necessary and sufficient to finish some certain computational tasks. In classical computation, a simplest model of computation is the decision tree (For more details, we can refer to the survey paper [1]). Correspondingly, the quantum query model (quantum black box model, or quantum decision tree model) is a generalization of the decision tree model in quantum computation [1,2,3,4,5]. Most of famous quantum algorithms are captured by the quantum query model [6], such as Shor’s factoring algorithm [7], Grover’s unstructured search algorithm [8], and so on [9,10,11]. The quantum query model can be investigated in the exact setting and the bounded-error setting [1]. Given an input $x\in D\subseteq {\left\{0,1\right\}}^{n}$ that can only be accessed through a black box by querying some bit $x_{i}$ of the input, the quantum query model computes an *n*-bit partial Boolean function f:D→{0,1} exactly (or with bounded-error) [1]. An exact quantum algorithm must always output the correct function value for all legal inputs [1]. If a quantum algorithm outputs the function value with a probability greater than a constant ($>\frac{1}{2}$) for all legal inputs, then the quantum algorithm is said to compute the function with bounded error. In the quantum query model, we care the quantum query complexity that is the decision tree complexity for the quantum model [1,2,3]. Roughly speaking, the exact (or bounded-error) quantum query complexity of a Boolean function denotes the number of queries of an optimal quantum decision tree that computes the Boolean function exactly (or with bounded-error) [1].

In quantum computation, the hope is to find out many problems whose computational complexity in quantum computer is less than the computational complexity in classical computer, i.e., finding out many problems that have the quantum advantage. For a function *f*, quantum advantage can be investigated by comparing the exact quantum query complexity $Q_{E}\left(f\right)$ and the deterministic decision tree complexity D(f) [1], where D(f) denotes the minimum number of queries used by any classical deterministic algorithm. Over the past decade, there have been many results on the quantum query model [12,13,14,15,16,17,18,19,20]. In particular, Ambainis et al. [14] proved that exact quantum algorithms have advantage for almost all Boolean functions in 2015. For total Boolean functions (i.e., partial Boolean functions with $D={\left\{0,1\right\}}^{n}$), the first known quantum speed-up was $Q_{E}\left(f\right)=O\left(D{\left(f\right)}^{0.8675\ldots }\right)$ by Ambainis [13], and then Ambainis et al. [21] presented a better separation with a quadratic gap between the exact quantum query complexity and the deterministic decision tree complexity, up to polylogarithmic factors.

For any partial Boolean function, the best separation is still achieved by Deutsch-Jozsa algorithm [10,17]. And, some main related results are as follows. In 2007, Montanaro [22] considered a problem of exact oracle identification with a single quantum query. In 2015, Montanaro et al. [15] investigated all small total Boolean functions up to four bits and symmetric total Boolean functions up to six bits. In 2016, Qiu et al. [17,23] generalized Deutsch-Jozsa problem and gave its optimal exact quantum query algorithm, and in particular, Qiu et al. [23,24] presented all symmetric partial Boolean functions (it is a special class of partial Boolean functions) with exact quantum query complexity 1, and proved that any symmetric partial Boolean function *f* can be computed exactly by a 1-query quantum algorithm if and only if *f* can be computed by the Deutsch-Jozsa algorithm [10]. In the same year, Aaronson et al. [25] showed an equivalence between 1-query quantum algorithms and bounded quadratic polynomials in the bounded-error setting. Also in the same year, Grillo et al. [26] investigated partial Boolean functions which are computed exactly with *t* queries. However, the result in [26] (i.e., Theorem 5) for any numerical procedure is difficult to use as an analytic tool. In 2017, Arunachalam et al. [27] proved a characterization of *t*-query quantum algorithms in terms of the unit ball of a space of degree-(2t) polynomials. Using the same method as the proof of Theorem 11 given by Qiu et al. [23,24], Chen et al. [28] showed that the total Boolean functions with exact quantum query complexity 1 are the one-bit function $f\left(x\right)=x_{1}$ and the two-bit function $x_{1}⊕x_{2}$, and Mukherjee et al. [29] noticed that this result of [28] is the same as a result by Montanaro et al. [15].

As above, the exact 1-query quantum model for all partial Boolean functions is expected to be investigated further. On one hand, similar to Refs. [23,24,25,26,27], establishing an equivalence between quantum query algorithms and some other theories will inspire more new algorithms and related results on the complexity theory. On the other hand, with the development of quantum computer, the problems solved by 1-query quantum algorithms may be the first to be used widely in the future, as the 1-query quantum algorithm costs the least unitary operators. Specifically, we investigate the following two problems.

- The partial Boolean functions can be regarded as a generalization of the total Boolean functions. Actually, Deutsch’s algorithm [9] computes a two-bit partial (also total) Boolean function using one query. Both the extension of Deutsch’s problem (computed by Deutsch-Jozsa algorithm [10]) and a generalized Deutsch-Jozsa problem in Ref. [17] are described by even *n*-bit partial (not total) Boolean functions. Naturally, what is the characterization of partial Boolean functions with exact quantum query complexity 1?
- In the field of quantum computation, it is a fundamental and interesting subject to evaluate the computational power of the 1-query quantum model, and is also critical for discovering quantum advantage. Specifically, the number of partial Boolean functions with exact quantum query complexity 1 shows the power and advantage of the exact 1-query quantum model. So, how many partial Boolean functions can be computed exactly by 1-query quantum algorithms?

The rest of the paper is organized as follows. In Section 2, we introduce some basis notations and the related knowledge. Then, we state and prove the first characterization and a related result in Section 3. Next, we state and prove the second characterization and two related results in Section 4. Finally, the conclusion is presented in Section 5. For the sake of brevity and readability, all proofs of lemmas in this paper are showed in Appendix A, Appendix B, Appendix C and Appendix D.

## 2. Preliminaries

In this section, we introduce some basic notations and recall some basic knowledge of partial Boolean functions and the exact quantum query model. For the details, we can refer to Refs. [1,6,20,23,30,31].

As usual, notations N, R, and C denote the sets of natural numbers, real numbers, and complex numbers, respectively. In particular, we will always use the notation *D* (or promised set) to denote a subset of ${\left\{0,1\right\}}^{n}$. For any input $x=x_{1}x_{2}\cdots x_{n}\in D$, the Hamming weight (number of 1s) of *x* is denoted by |x|. Given a real number set *S*, the notation maxS denotes the maximum in *S* and the notation minS denotes the minimum in *S*. For any finite set *S*, the notation |S| denotes the number of elements in *S*. For a complex matrix *A*, $A^{T}$ is the transpose of the matrix *A*, and $A^{†}={\left(A^{T}\right)}^{*}$ is the conjugate transpose of the matrix *A*. Obviously, $A^{†}=A^{T}$ for any real matrix *A*. Furthermore, the notation |a〉 is usually used to denote a quantum state which is a unit vector in a Hilbert space and labeled by the notation *a*. In particular, ${\langle a|=(|a\rangle )}^{†}$ is a row vector.

In this paper, we mainly concern partial functions f:D→C, f:D→R and f:D→{0,1}. In general, these functions can be given by a $2^{n}$-dimensional vector (f(0),f(1),⋯,f(x),$\cdots ,f\left(2^{n}-1\right){)}^{T}$ whose entry f(x) is denoted by * for any undefined input $x\in {\left\{0,1\right\}}^{n}\setminus D$. For example, the Boolean function *f* computed by Deutsch’s algorithm [9] can be given by (f(00),f(01),f(10),f(11))=(1,0,0,1). Sometimes, we also use a two-tuple ({x:f(x)=0},{y:f(y)=1}) to give a certain partial Boolean function f:D→{0,1}. For example, the even *n*-bit partial Boolean function *f* computed by Deutsch-Jozsa algorithm [10] can be given by $(\{x\in {\left\{0,1\right\}}^{n}:|x|=0,n\},\{y\in {\left\{0,1\right\}}^{n}:|y|=\frac{n}{2}\})$.

In order to represent these functions, we need to use two monomials $X_{S}=\prod_{i\in S}x_{i}$ and ${\left(-1\right)}^{S\cdot x}=\prod_{i\in S}{\left(-1\right)}^{x_{i}}$ [1,20,30]. In particular, $X_{Ø}={\left(-1\right)}^{Ø\cdot x}=1$. And, the set $\left\{X_{S}:S\subseteq \left\{1,2,\cdots ,n\right\}\right\}$ is usually called the polynomial basis and the set $\left\{{\left(-1\right)}^{S\cdot x}:S\subseteq \left\{1,2,\cdots ,n\right\}\right\}$ is usually called Fourier basis [1,20,30]. If a function $p:R^{n}\to C$ can be written as $\Sigma_{S}\alpha_{S}X_{S}$ for some complex numbers $\alpha_{S}$, then the function *p* is called a multilinear polynomial [1]. Meanwhile, the degree of the multilinear polynomial *p* is defined by $\deg\left(p\right)=\max\{|S|:\alpha_{S}\neq 0\}$. For any partial function f:D→C, a multilinear polynomial p(x) represents *f* if and only if p(x)=f(x) for all x∈D [1,32]. Unlike total functions $f:{\left\{0,1\right\}}^{n}\to C$, the multilinear representation of a partial (not total) function f:D→C is usually not unique. Naturally, the degree of a partial (or total) function f:D→C can be defined by deg(f)=min{deg(p):prepresentsf}.

In the quantum query model, for every input x∈D, the quantum black box $O_{x}$ can be described as a unitary operator which is defined by

(1) $$O_{x}|i,j\rangle =\left\{\begin{array}{ccc} {\left(-1\right)}^{x_{i}}|i,j\rangle , & \; & \mathrm{if}\;\;i\in \{1,2,\cdots ,n\},\\ |0,j\rangle , & \; & \mathrm{if}\;\;i=0. \end{array}\right.$$

Here, the integer number i∈{0,1,2,⋯,n} is the query-part and the label *j* is the auxiliary space. As a result, a *t*-query quantum algorithm can be determined by an initial state $|\psi_{0}\rangle$ and a sequence of unitary transformations $U_{0},O_{x},U_{1},O_{x},\cdots ,O_{x},U_{t}$ followed by a measurement, where t+1 unitary operators $U_{0},U_{1},\cdots ,U_{t}$ are independent of the input [1,6].

## 3. The First Characterization

This section introduces and proves the first characterization and a related result.

### 3.1. A Characterization by the Linear System of Equations

Inspired by proofs of Theorem 8 in Ref. [23], the first characterization is presented in the following. In fact, the characterization can also be got by Theorem 5 in [26].

**Theorem** **1.** *An n-bit non-constant partial Boolean function f:D→{0,1} can be computed exactly by a 1-query quantum algorithm, if and only if there exist at least one non-negative solution z=($z_{0},$$z_{1},$$z_{2},$⋯,$z_{n}{)}^{T}$ of equations $z_{0}+$$z_{1}+$$z_{2}+$⋯+$z_{n}$=1 and $F^{T}\left(a⊕b\right)z=0$ for all a∈{x:f(x)=0} and b∈{x:f(x)=1} where the function vector $F\left(x\right)={\left(1,{\left(-1\right)}^{x_{1}},{\left(-1\right)}^{x_{2}},\cdots ,{\left(-1\right)}^{x_{n}}\right)}^{T}$ for any $x=x_{1}x_{2}\cdots x_{n}\in {\left\{0,1\right\}}^{n}$.*

**Proof.**  ⇒). Since the algorithm is exact, the quantum state $U_{1}O_{a}U_{0}|\psi_{0}\rangle$ for all a∈{x:f(x)=0} must be orthogonal to the quantum state $U_{1}O_{b}U_{0}|\psi_{0}\rangle$ for all b∈{x:f(x)=1}. Meanwhile, since the unitary operator $U_{1}$ preserves the inner product of any two complex vectors, the quantum state $O_{a}U_{0}|\psi_{0}\rangle$ for all a∈{x:f(x)=0} must be orthogonal to the quantum state $O_{b}U_{0}|\psi_{0}\rangle$ for all b∈{x:f(x)=1}. For any quantum state $U_{0}|\psi_{0}\rangle =\sum_{i,j}\alpha_{i,j}|i,j\rangle ,$ note that

(2) $$O_{a}U_{0}|\psi_{0}\rangle =\sum_{j}\alpha_{0,j}|0,j\rangle +\sum_{i,j}\alpha_{i,j}{\left(-1\right)}^{a_{i}}|i,j\rangle$$

and the inner product

(3) $$\begin{array}{cc} \; & (O_{a}U_{0}|\psi_{0}\rangle {)}^{†}O_{b}U_{0}|\psi_{0}\rangle =\sum_{j}|\alpha_{0,j}{|}^{2}+\sum_{i,j}{\left(-1\right)}^{a_{i}⊕b_{i}}{\left|\alpha_{i,j}\right|}^{2}\\ = & (\sum_{j}|\alpha_{0,j}{|}^{2},\sum_{j}|\alpha_{1,j}{|}^{2},\cdots ,\sum_{j}|\alpha_{n,j}{|}^{2}){\left(1,{\left(-1\right)}^{a_{1}⊕b_{1}},\cdots ,{\left(-1\right)}^{a_{n}⊕b_{n}}\right)}^{T}\\ = & \left(z_{0},z_{1},\cdots ,z_{n}\right)F\left(a⊕b\right)=z^{T}F\left(a⊕b\right) \end{array}$$

where the notation $z_{i}=\sum_{j}{\left|\alpha_{i,j}\right|}^{2}$ for all i∈{0,1,2,⋯,n} is introduced in the last equality. Thus, there exists at least one non-negative solution z=($z_{0},$$z_{1},$$z_{2},$⋯,$z_{n}{)}^{T}$ of equations $z_{0}+$$z_{1}+$$z_{2}+$⋯+$z_{n}$= 1 and $z^{T}F\left(a⊕b\right)=0$ for all a∈{x:f(x)=0} and b∈{x:f(x)=1}.

⇐). For a non-negative solution z=($z_{0},$$z_{1},$$z_{2},$⋯,$z_{n}{)}^{T}$ of equations $z_{0}+$$z_{1}+$$z_{2}+$⋯+$z_{n}$= 1 and $z^{T}F\left(a⊕b\right)=0$ for all a∈{x:f(x)=0} and b∈{x:f(x)=1}, if we set $\sum_{j}{\left|\alpha_{i,j}\right|}^{2}=z_{i}$ for all i∈{0,1,2,⋯,n} in the state $U_{0}|\psi_{0}\rangle =\sum_{i,j}\alpha_{i,j}|i,j\rangle$ of an undetermined 1-query quantum algorithm, then the inner product

(4) $$\begin{array}{c} (U_{1}O_{a}U_{0}|\psi_{0}\rangle {)}^{†}U_{1}O_{b}U_{0}|\psi_{0}\rangle =0 \end{array}$$

for all a∈{x:f(x)=0} and b∈{x:f(x)=1}. By Gram-Schmidt orthogonalization, we can find an orthonormal basis $\{U_{1}O_{a}U_{0}|\psi_{0}\rangle :f\left(a\right)=0,a\in D\}$ and an orthonormal basis $\{U_{1}O_{b}U_{0}|\psi_{0}\rangle :f\left(b\right)=1,b\in D\}$, respectively. By the measurement consisting of the two orthonormal bases, the 1-query quantum algorithm (with the state $U_{0}|\psi_{0}\rangle =\sum_{i,j}\alpha_{i,j}|i,j\rangle$) computes *f* exactly. Thus, Theorem 1 has been proved. □

#### Discussions on Theorem 1

Theorem 1 transforms the problem of deciding the partial Boolean function with exact quantum query complexity 1 into the problem of solving a linear system of equations. Specifically, the number of variables in the linear system is n+1 and the number of equations is $1+\left|\left\{a⊕b:f\left(a\right)=0,f\left(b\right)=1\right\}\right|\leq 2^{n}$.

Considering the definition of the exact quantum query complexity, the task of proving $Q_{E}\left(f\right)=k$ must be finished by presenting an exact *k*-query quantum algorithm. For a small *n*, presenting an optimal exact quantum query algorithm is still quite hard in some cases. For k=1, Theorem 1 transforms this task to the problem of solving a linear system which is a computable task. For any non-constant *n*-bit partial Boolean function f:D→{0,1}, if |D| is not big, then this task can be done efficiently in a classical computer. This is the most important contribution of Theorem 1.

In fact, Theorem 1 is also a practical tool in some cases. In the worst case, the number of equations in the linear system is 1+|{a⊕b:f(a)=0,f(b)=1}| which is an exponential number. Note that the number of variables in the linear system is n+1. So, the following two results can be got.

(1) Given any *n*-bit partial Boolean function *f*, if some equations (By basic linear algebra, n+1 equations are enough in the best case) lead to an empty (non-negative real) solution of the linear system, then by Theorem 1 these equations are enough to prove that the exact quantum query complexity of *f* is bigger than 1. Thus, for the best case, other |{a⊕b:f(a)=0,f(b)=1}|−n equations can be ignored and things become quite easy.
(2) If the exact quantum query complexity of an *n*-bit partial Boolean function f:D→{0,1} is bigger than 1, then the exact quantum query complexity of any *n*-bit partial Boolean function *g* defined by
  (5) $$\left\{\begin{array}{cc} g(x)=f(x), & \forall x\in D,\\ g(x)\in \{0,1,*\}, & \mathrm{otherwise} \end{array}\right.$$
  is also bigger than 1. The number of partial Boolean functions in this form is $3^{2^{n}-\left|D\right|}$ which is also an exponential number.

### 3.2. Partial Boolean Functions Depending on All Bits

This subsection considers a special class of all partial Boolean functions using Theorem 1.

First, we introduce the following definition [14,31] (A background of this definition is introduced briefly in Appendix A).

**Definition** **1.** *[14,31]. An n-bit partial Boolean function f:D→{0,1} is said to depend on k (≤n) bits, if k is the minimum number of variables in all multilinear polynomials representing f.*

By Definition 1, the two-bit total Boolean function computed by Deutsch’s algorithm [9] depends on two bits, and, for even *n*, the *n*-bit partial Boolean function computed by Deutsch-Jozsa algorithm [10] depends on $\frac{n}{2}+1$ bits.

For partial Boolean functions depending on all bits, the result is stated in the following.

**Theorem** **2.** *For any n-bit partial Boolean function f:D→{0,1} depending on all n bits, f can be computed exactly by a 1-query quantum algorithm, if and only if $f\left(x\right)=x_{1}$ or $1⊕x_{1}$ with D={0,1}, or $f\left(x\right)=x_{1}⊕x_{2}$ or $1⊕x_{1}⊕x_{2}$ with $D\in \{E\subseteq {\left\{0,1\right\}}^{2}:|E|\in \left\{3,4\right\}\}$. □*

**Proof.**  ⇒). For any *n*-bit partial Boolean function f:D→{0,1} and k∈{1,2,⋯,n}, any multilinear polynomial representation of *f* can be written as

(6) $$f\left(x\right)=x_{k}q_{1}\left(x_{1},x_{2},\cdots ,x_{k-1},x_{k+1},\cdots ,x_{n}\right)+q_{2}\left(x_{1},x_{2},\cdots ,x_{k-1},x_{k+1},\cdots ,x_{n}\right)$$

where $q_{1}\left(x_{1},x_{2},\cdots ,x_{k-1},x_{k+1},\cdots ,x_{n}\right)$ and $q_{2}\left(x_{1},x_{2},\cdots ,x_{k-1},x_{k+1},\cdots ,x_{n}\right)$ are two multilinear polynomials on variables $x_{1},x_{2},\cdots ,x_{k-1},x_{k+1},\cdots ,x_{n}$ (i.e., not on the variable $x_{k}$). Let the input $X_{k}^{\left\{k\right\}}$ be the same as the input $X_{k}$ except for the *k*-th bit being flipped. With an argument, if *f* depends on *n* bits (By Definition 1, this means that the number of variables in all multilinear polynomials representing *f* is at least *n*), then there must exist at least *n* input pairs $\left(X_{1},X_{1}^{\left\{1\right\}}\right)$, $\left(X_{2},X_{2}^{\left\{2\right\}}\right)$, ⋯,$\left(X_{n},X_{n}^{\left\{n\right\}}\right)$ such that $1⊕f\left(X_{k}\right)=f\left(X_{k}^{\left\{k\right\}}\right)\in \left\{0,1\right\}$ for all k∈{1,2,⋯,n} (In fact, for a certain *k*, if $f\left(X\right)=f\left(X^{\left\{k\right\}}\right)$ always hold for any X∈D, then the polynomial $q_{1}\left(x_{1},x_{2},\cdots ,x_{k-1},x_{k+1},\cdots ,x_{n}\right)=0$ and $f\left(x\right)=q_{2}\left(x_{1},x_{2},\cdots ,x_{k-1},x_{k+1},\cdots ,x_{n}\right)$ depends on at most n−1 bits. This result contradicts the assumption that *f* depends on *n* bits).

By Theorem 1, there exists a non-negative vector z=($z_{0},$$z_{1},$$z_{2},$⋯,$z_{n}{)}^{T}$ such that the equations $z_{0}+$$z_{1}+$$z_{2}+$⋯+$z_{n}$=1 and

(7) $$z^{T}F\left(X_{k}⊕X_{k}^{\left\{k\right\}}\right)=0=z_{0}+\sum_{i\neq k}z_{i}-z_{k},k\in \left\{1,2,\cdots ,n\right\}$$

hold. Combining with $z_{0}+z_{1}+z_{2}+\cdots +z_{n}=1=z_{0}+\sum_{i\neq k}z_{i}+z_{k}$, we have $2z_{k}=1$ for all k∈{1,2,⋯,n} which implies that n=1 with $z={\left(\frac{1}{2},\frac{1}{2}\right)}^{T}$ or n=2 with $z={\left(0,\frac{1}{2},\frac{1}{2}\right)}^{T}$.

The case n=1 is trivial, and *f* can be given by (f(0),f(1))=(0,1) or (1,0). For the case n=2, the unique non-negative solution $z={\left(0,\frac{1}{2},\frac{1}{2}\right)}^{T}$ implies that

(8) $$\frac{1}{2}{\left(-1\right)}^{a_{1}⊕b_{1}}+\frac{1}{2}{\left(-1\right)}^{a_{2}⊕b_{2}}=0$$

for all a∈{x:f(x)=0} and b∈{x:f(x)=1}. Then, $a_{1}⊕a_{2}\neq b_{1}⊕b_{2}$ for all a∈{x:f(x)=0} and b∈{x:f(x)=1}. This result implies that $f\left(x\right)=x_{1}⊕x_{2}$ or $1⊕x_{1}⊕x_{2}$. Meanwhile, since f:D→{0,1} is a two-bit partial Boolean function depending on two bits, |D|∈{3,4}.

⇐). This direction is trivial. Thus, Theorem 2 has been proved. □

#### Discussions on Theorem 2

Since there are many partial Boolean functions with exact quantum query complexity 1, it is necessary to divide them into some classes. In 2015, Montanaro et al. [15] investigated all small total Boolean functions up to four bits and symmetric total Boolean functions up to six bits. In 2016, Qiu et al. [23,24] studied all symmetric partial Boolean functions and remained others open. By Definition 1, it is natural to divide all *n*-bit partial Boolean functions into n+1 classes.

For all *n*-bit partial Boolean functions depending on 1 and 2 bits, the problem is trivial. For *n*-bit partial Boolean functions depending on *n* bits, the result is put into Theorem 2. By a trivial (not direct) argument, the statement that a partial Boolean function *f* depends on all *n* bits is consistent with previous definition. This is a key hint in the proof. Intuitively, this implication seems obvious. However, since the implication does not hold for $\frac{n}{2}$, we remark that a proof is necessary.

Theorem 2 clarifies all partial Boolean functions that depend on all bits and can be computed exactly by a 1-query quantum algorithm. Observing the unique multilinear polynomial of any total Boolean function, any *n*-bit total Boolean function depending on *k*(≤n) bits can be identified with a *k*-bit total Boolean function depending on *k* bits. Therefore, Theorem 2 generalizes the result on total Boolean functions [15] to partial Boolean functions. Surprisingly, the number of all *n*-bit partial Boolean functions depending on *n* bits is quite big. This fact is implied by the following lemma.

**Lemma** **1.** *Let N(n)(n≥1) denote the number of all n-bit partial Boolean functions depending on n bits. Then, $N\left(n\right)\geq 2\times 3^{2^{n}-n-1}.$*

In contrast, the number of all *n*-bit total Boolean functions (investigated by Montanaro et al. [15]) is $2^{2^{n}}$ and the number of all *n*-bit symmetric partial Boolean functions (investigated by Qiu et al. [23,24]) is $3^{n+1}$.

As a result, many *n*-bit partial Boolean functions depending on k∈{3,⋯,n−1} bits is sitll unclear. This motivate us to investigate partial Boolean functions further.

## 4. The Second Characterization

This section introduces and proves the second characterization and two related results.

### 4.1. A Characterization by the Sum-of-Squares Representation

Following the discussions of Lemma 7 and Theorem 17 in [1], if f:D→{0,1} can be computed by an exact 1-query quantum algorithm, then there exist degree-1 SOS complex representations of *f* and 1−f. Thus, this subsection introduces and proves a characterization using the SOS representation.

First, the following lemma follows the discussions of Lemma 7 and Theorem 17 in [1]. This lemma is proved and also used in our recent paper [33].

**Lemma** **2.** *[1]. If there exists an exact 1-query quantum algorithm computing an n-bit partial Boolean function f:D→{0,1}, then there must exist degree-1 SOS complex (multilinear polynomials) representations of f and 1−f.*

In order to give the second characterization, we introduce the following definition. This definition is also used in [33]. More related definitions can be seen in Refs. [34,35,36,37,38,39].

**Definition** **2.** *[34,35,36,37,38,39]. Let the function vector $F\left(x\right)={\left(1,{\left(-1\right)}^{x_{1}},{\left(-1\right)}^{x_{2}},\cdots ,{\left(-1\right)}^{x_{n}}\right)}^{T}$. For an n-bit partial Boolean function f:D→{0,1}, if there exist two real (1+n)-dimensional column vector sets $\left\{a_{1},a_{2},\cdots ,a_{p}\right\}$ and $\left\{a_{p+1},a_{p+2},\cdots ,a_{p+q}\right\}$ such that the equations*

(9) $$\left\{\begin{array}{cc} f\left(x\right)=\sum_{l=1}^{p}{\left|a_{l}^{T}F\left(x\right)\right|}^{2}, & x\in D,\\ 1-f\left(x\right)=\sum_{l=p+1}^{p+q}{\left|a_{l}^{T}F\left(x\right)\right|}^{2}, & x\in D,\\ \sum_{l=p+1}^{p+q}|a_{l}^{T}{F\left(x\right)|}^{2}=1-\sum_{l=1}^{p}{\left|a_{l}^{T}F\left(x\right)\right|}^{2}, & x\in {\left\{0,1\right\}}^{n}, \end{array}\right.$$

*hold, then the (p+q)×(1+n) complex coefficient matrix $\left[\alpha_{f}\right]$ in the form of*

(10) $$\left[\alpha_{f}\right]=\left[\begin{array}{c} \begin{array}{c} a_{1}^{T}\\ \vdots \\ a_{p}^{T}\\ a_{p+1}^{T}\\ \vdots \\ a_{p+q}^{T} \end{array} \end{array}\right]$$

*is called a degree-1 SOS complex representation matrix of f and 1−f. Here, every row vector $a_{p}^{T}$ is the coefficient vector of the degree-1 Fourier polynomial $a_{l}^{T}F\left(x\right)$ in Equation (9).*

As we know, for the state $U_{1}O_{x}U_{0}|\psi_{0}\rangle =\sum_{i,j}\left(\sum_{S}\alpha_{i,j}^{S}{\left(-1\right)}^{S\cdot x}\right)|i,j\rangle$ in a 1-query quantum algorithm, the amplitude $\sum_{S}\alpha_{i,j}^{S}{\left(-1\right)}^{S\cdot x}$ of any basis state |i,j〉 is a polynomial of degree ≤1. Therefore, $\sum_{S}\alpha_{i,j}^{S}{\left(-1\right)}^{S\cdot x}=\left(\alpha_{i,j}^{Ø},\alpha_{i,j}^{\left\{1\right\}},\alpha_{i,j}^{\left\{2\right\}},\cdots ,\alpha_{i,j}^{\left\{n\right\}}\right){\left(1,{\left(-1\right)}^{x_{1}},{\left(-1\right)}^{x_{2}},\cdots ,{\left(-1\right)}^{x_{n}}\right)}^{T}$. Then, the coefficient matrix $\left[\alpha_{i,j}^{S}\right]$ (here, the number pair i,j is the row index and the number set *S* the column index. In a 1-query quantum algorithm, the number pair i,j traverses all basis states and the set *S* traverses Ø,{1},{2},⋯,{n}) with n+1 columns in the form of

(11) $$\left[a_{Ø},a_{\left\{1\right\}},a_{\left\{2\right\}},\cdots ,a_{\left\{n\right\}}\right]$$

can be used to represent the state $U_{1}O_{x}U_{0}|\psi_{0}\rangle$. Without loss of generality, assume that the basis states in a 1-query quantum algorithm are the computational basis {|0〉,|1〉,|2〉,⋯}. Next, the equation $U_{1}O_{x}U_{0}|\psi_{0}\rangle =\left[\alpha_{i,j}^{S}\right]$F(x) holds (here, $F\left(x\right)={\left(1,{\left(-1\right)}^{x_{1}},{\left(-1\right)}^{x_{2}},\cdots ,{\left(-1\right)}^{x_{n}}\right)}^{T}$). In order to distinguish the coefficient matrix of the state $O_{x}U_{0}|\psi_{0}\rangle$ from the coefficient matrix of the state $U_{1}O_{x}U_{0}|\psi_{0}\rangle$, the notation $\left[\beta_{i,j}^{S}\right]$ denotes the coefficient matrix $U_{1}^{-1}\left[\alpha_{i,j}^{S}\right]$ of the state $O_{x}U_{0}|\psi_{0}\rangle$.

By Definition 2 and the coefficient matrix, the second characterization is presented in the following.

**Theorem** **3.** *Any n-bit non-constant partial Boolean function f:D→{0,1} can be computed exactly by a 1-query quantum algorithm, if and only if there exists a degree-1 SOS complex representation matrix $\left[\alpha_{f}\right]$ of f and 1−f such that*

(12) $${\left[\alpha_{f}\right]}^{†}\left[\alpha_{f}\right]=\mathrm{diag}\left(u_{0},u_{1},u_{2},\cdots ,u_{n}\right).$$

**Proof.**  ⇒). On one hand, the coefficient matrices of the states $U_{1}O_{x}U_{0}|\psi_{0}\rangle$ and $O_{x}U_{0}|\psi_{0}\rangle$ are in the form of

(13) $$\left[\alpha_{i,j}^{S}\right]=\left[a_{Ø},a_{\left\{1\right\}},a_{\left\{2\right\}},\cdots ,a_{\left\{n\right\}}\right]$$

and

(14) $$\left[\beta_{i,j}^{S}\right]=U_{1}^{-1}\left[\alpha_{i,j}^{S}\right],$$

respectively. On the other hand, for any quantum state

(15) $$U_{0}|\psi_{0}\rangle =\sum_{j}\alpha_{0,j}^{Ø}|0,j\rangle +\sum_{i\neq 0}\sum_{j}\alpha_{i,j}^{Ø}|i,j\rangle ,$$

the state

(16) $$O_{x}U_{0}|\psi_{0}\rangle =\sum_{j}\alpha_{0,j}^{Ø}|0,j\rangle +\sum_{i\neq 0}\sum_{j}\alpha_{i,j}^{Ø}{\left(-1\right)}^{x_{i}}|i,j\rangle .$$

Thus, the coefficient matrix of the state $O_{x}U_{0}|\psi_{0}\rangle$ is in the form of a block diagonal matrix

(17) $$\left[\beta_{i,j}^{S}\right]=\mathrm{diag}\left(B_{0},B_{1},\cdots ,B_{n}\right)$$

where the *i*-th block matrix

(18) $$B_{i}=\left[\begin{array}{c} \begin{array}{c} \alpha_{i,0}^{Ø}\\ \alpha_{i,1}^{Ø}\\ \vdots \\ \alpha_{i,j}^{Ø}\\ \vdots \end{array} \end{array}\right]$$

for every fixed i∈{0,1,2,⋯,n}. Thus, $U_{1}^{-1}$$\left[\alpha_{i,j}^{S}\right]=\left[\beta_{i,j}^{S}\right]=\mathrm{diag}(B_{0},$$B_{1},$⋯,$B_{n}).$

According to Lemma 2 and Definition 2, the coefficient matrix $\left[\alpha_{i,j}^{S}\right]$ of the state $U_{1}O_{x}U_{0}|\psi_{0}\rangle$ is an SOS complex representation matrix $\left[\alpha_{f}\right]$ of the partial Boolean function *f* and 1−f. Meanwhile, since all columns of any block-diagonal matrix $\mathrm{diag}(B_{0},$$B_{1},$⋯,$B_{n})$ are pairwise orthogonal and the unitary operator $U_{1}^{-1}$ preserves the inner product of any two complex vectors, all columns of the matrix $\left[\alpha_{f}\right]$ are also pairwise orthogonal. Thus, Equation (12) holds.

⇐). For a degree-1 SOS complex representation matrix $\left[\alpha_{f}\right]$ of *f* and 1−f satisfying ${\left[\alpha_{f}\right]}^{†}\left[\alpha_{f}\right]$=$\mathrm{diag}(u_{0},$$u_{1},$$u_{2},$⋯,$u_{n})$, all columns of the matrix $[\alpha_{f}]=$$\left[a_{Ø},a_{\left\{1\right\}},a_{\left\{2\right\}},\cdots ,a_{\left\{n\right\}}\right]$ are pairwise orthogonal. Note that we can always get a sequence of proper vectors $B_{0},$$B_{1},$⋯,$B_{n}$ satisfying $\left|\right|B_{0}\left|\right|$=$\left|\right|a_{Ø}\left|\right|=\sqrt{u_{0}}$ and $\left|\right|B_{i}\left|\right|$=$\left|\right|a_{\left\{i\right\}}\left|\right|=\sqrt{u_{i}}$ for all i∈{1,2,⋯,n} (for example, $B_{i}={\left(\sqrt{u_{i}},0,0,\cdots \right)}^{T}$). Since all columns of $\left[\alpha_{f}\right]$ and $\mathrm{diag}(B_{0},$$B_{1},$⋯,$B_{n})$ are pairwise orthogonal, there always exists a unitary operator $U_{1}^{-1}$ such that $U_{1}^{-1}$$\left[\alpha_{f}\right]$=$\mathrm{diag}(B_{0},$$B_{1},$⋯,$B_{n})$.

As a result, the three states $U_{1}O_{x}U_{0}|\psi_{0}\rangle$, $O_{x}U_{0}|\psi_{0}\rangle$ and $U_{0}|\psi_{0}\rangle$ of an exact 1-query quantum algorithm computing *f* can be determined by $\left[\alpha_{f}\right]$, $\mathrm{diag}(B_{0},$$B_{1},$⋯,$B_{n})$ and

(19) $$\left[\begin{array}{c} \begin{array}{c} B_{0}\\ B_{1}\\ \vdots \\ B_{n} \end{array} \end{array}\right],$$

respectively. Thus, Theorem 3 has been proved. □

#### Discussions on Theorem 3

In order to use Theorem 3, we need to find a pair of SOS real representations of *f* and 1−f first, and then transform it into a proper SOS complex representation matrix. Since it is feasible to get a pair of SOS real representations for very small (partial) Boolean functions [34,35,36,37,38,39], Theorem 3 can be tested on very small partial Boolean functions.

To some extent, Theorem 3 provides a different style for the characterization of partial Boolean function with exact quantum query complexity 1. On one hand, similar to Ref. [25] (showed an equivalence between 1-query quantum algorithms and bounded quadratic polynomials in the bounded-error setting) and [27] (proved a characterization of *t*-query quantum algorithms in terms of the unit ball of a space of degree-(2t) polynomials), Theorem 3 shows an equivalence between the sum-of-squares polynomial representations and the exact 1-query quantum algorithm. In fact, Theorem 3 transforms the problem of proving $Q_{E}\left(f\right)=1$ to the problem of solving a system of multivariate quadratic equations which is a difficult problem in practical applications. On the other hand, combining Theorem 3 with Theorem 1, we can see that the problem of solving the system of multivariate quadratic equations in Theorem 3 can be reduced to the problem of solving the linear system of equations in Theorem 1. This result is a quantum-inspired result which is an interesting application of the quantum theory.

### 4.2. Partial Boolean Functions Depending on *k* Bits

This subsection considers partial Boolean functions depending on *k* bits.

Inspired by Theorem 3, we get the following result.

**Theorem** **4.** *Let the function vector $P\left(x\right)={\left(1,x_{1},x_{2},\cdots ,x_{n}\right)}^{T}$ where $x=x_{1}x_{2}\cdots x_{n}\in {\left\{0,1\right\}}^{n}$. For any n-bit non-constant partial Boolean function f:D→{0,1}, if f depends on k bits and can be computed exactly by a 1-query quantum algorithm, then*

(20) $$\mathrm{rank}\left({\left(\cdots ,P\left(x\right),\cdots \right)}_{f(x)=0}\right),\mathrm{rank}\left({\left(\cdots ,P\left(x\right),\cdots \right)}_{f(x)=1}\right)\in \left\{1,2,\cdots ,n\right\}$$

*and*

(21) $$\mathrm{rank}\left({\left(\cdots ,P\left(x\right),\cdots \right)}_{f(x)=0}\right)+\mathrm{rank}\left({\left(\cdots ,P\left(x\right),\cdots \right)}_{f(x)=1}\right)-\left(2n+2-k\right)\leq 0$$

*where all columns P(x) in the matrix ${\left(\cdots ,P\left(x\right),\cdots \right)}_{f(x)=b}$ traverse all legal inputs x∈D satisfying f(x)=b for b∈{0,1}.*

In order to prove Theorem 4, the following lemma is necessary.

**Lemma** **3.** *If an n-bit partial Boolean function f:D→{0,1} depends on k (≤n) bits and there exists a degree-1 SOS complex representation of f, then there exist at least k non-zero columns $a_{\left\{i_{1}\right\}}$, $a_{\left\{i_{2}\right\}}$, ⋯,$a_{\left\{i_{k}\right\}}$ in the matrix $\left[\alpha_{f}\right]$ where $i_{1},$$i_{2},$⋯,$i_{k}\in \left\{1,2,\cdots ,n\right\}$.*

Then, Theorem 4 can be proved in the following.

**Proof.**  Recall that all columns of the matrix ${\left(\cdots ,P\left(x\right),\cdots \right)}_{f(x)=b}$ traverse all legal inputs x∈D satisfying f(x)=b where the vector function $P\left(x\right)={\left(1,x_{1},x_{2},\cdots ,x_{n}\right)}^{T}$. Note that the size of the matrix ${\left(\cdots ,P\left(x\right),\cdots \right)}_{f(x)=b}$ is (n+1)×|{x:f(x)=b}|.

On one hand, for a non-constant *n*-bit partial Boolean function *f*, if there exists a degree-1 SOS complex representation, then there exists a sequence of (n+1)-dimensional non-zero vectors $a_{1}$, $a_{2}$, ⋯,$a_{p}$ satisfying

(22) $$f\left(x\right)=\sum_{l=1}^{p}{\left|a_{l}^{T}P\left(x\right)\right|}^{2},\forall x\in D.$$

Considering Equation (22) for *x* such that f(x)=0 forces $a_{l}^{T}P\left(x\right)=0$. Thus, $a_{l}⏊P\left(x\right)$ for all P(x) with f(x)=0. Then, $a_{l}$ for any *l* is in the orthogonal complement of the space spanned by all P(x) with f(x)=0, we know the existence of the sequence (i.e., non-zero vectors $a_{1}$, $a_{2}$, ⋯,$a_{p}$) requires 1≤(n+1)−$\mathrm{rank}({\left(\cdots ,P\left(x\right),\cdots \right)}_{f(x)=0})$≤n. Similarly, considering Equation (22) for *x* such that f(x)=1, we can get 1≤(n+1)$-\mathrm{rank}({\left(\cdots ,P\left(x\right),\cdots \right)}_{f(x)=1})$≤n. Thus, Equation (20) holds.

On the other hand, by Theorem 3, for an *n*-bit partial Boolean function *f* with exact quantum query complexity 1, there exists an SOS complex representations matrix $\left[a_{Ø},a_{\left\{1\right\}},a_{\left\{2\right\}},\cdots ,a_{\left\{n\right\}}\right]=\left[\alpha_{f}\right]$ of *f* and 1−f such that $U_{1}^{-1}$$\left[\alpha_{f}\right]$$=\mathrm{diag}(B_{0},$$B_{1},$⋯,$B_{n}).$ By Equation (10), we can see that

(23) $$\mathrm{rank}\left(\left[\alpha_{f}\right]\right)\leq \mathrm{rank}\left(\left[a_{1},\cdots ,a_{p}\right]\right)+\mathrm{rank}\left(\left[a_{p+1},\cdots ,a_{p+q}\right]\right).$$

Moreover,

(24) $$\mathrm{rank}\left(\left[a_{1},\cdots ,a_{p}\right]\right)\leq \left(1+n\right)-\mathrm{rank}\left({\left(\cdots ,P\left(x\right),\cdots \right)}_{f(x)=0}\right)$$

and

(25) $$\mathrm{rank}\left(\left[a_{p+1},\cdots ,a_{p+q}\right]\right)\leq \left(1+n\right)-\mathrm{rank}\left({\left(\cdots ,P\left(x\right),\cdots \right)}_{f(x)=1}\right)$$

can be obtained by considering Equation (22) for *x* such that f(x)=0 and f(x)=1, respectively. Using the property (i.e., preserving Euclidean norm and the rank) of the unitary matrix and Lemma 3,

(26) $$\begin{array}{cc} k\leq & |\{i\in \left\{0,1,\cdots ,n\right\}:a_{\left\{i\right\}}\neq 0\}|\;\;\;\;\left(\mathrm{Lemma}\;3\right)\\ = & |\{i\in \left\{0,1,\cdots ,n\right\}:∥B_{i}∥\neq 0\}|\;\;\;\;\left(\mathrm{The}\;\mathrm{unitary}\;\mathrm{operator}\;U_{1}\;\mathrm{preserves}\;\mathrm{Euclidean}\;\mathrm{norm}\right)\\ = & \mathrm{rank}(\mathrm{diag}\left(B_{0},B_{1},\cdots ,B_{n}\right))\;\;\;\;\left(\mathrm{The}\;\mathrm{characterization}\;\mathrm{of}\;\mathrm{the}\;\mathrm{block}\;\mathrm{diagonal}\;\mathrm{matrix}\right)\\ = & \mathrm{rank}\left(\left[a_{Ø},a_{\left\{1\right\}},a_{\left\{2\right\}},\cdots ,a_{\left\{n\right\}}\right]\right)\;\;\;\;\left(\mathrm{The}\;\mathrm{unitary}\;\mathrm{operator}\;U_{1}\;\mathrm{preserves}\;\mathrm{the}\;\mathrm{rank}\right)\\ = & \mathrm{rank}\left(\left[\alpha_{f}\right]\right)\leq 2\left(1+n\right)-\sum_{b=0}^{1}\mathrm{rank}\left({\left(\cdots ,P\left(x\right),\cdots \right)}_{f(x)=b}\right).\;\;\;\;\left(\mathrm{Equations}\;(23)–(25)\right) \end{array}$$

Thus, Theorem 4 has been proved. □

#### Discussions on Theorem 4

The inverse direction of Theorem 4 is not always hold. For example, a three-bit partial Boolean function *f* given by the two-tuple $(\{x\in {\left\{0,1\right\}}^{3}:|x|=0\},\{y\in {\left\{0,1\right\}}^{3}:|y|=1\})$. Here, it is not difficult to know that $\mathrm{rank}({\left(\cdots ,P\left(x\right),\cdots \right)}_{f(x)=0})=1$ and $\mathrm{rank}({\left(\cdots ,P\left(x\right),\cdots \right)}_{f(x)=1})=3$. However, using Theorem 10 of Ref. [23], $Q_{E}\left(f\right)\geq 2$.

Theorem 4 gives a necessary condition on the case that an *n*-bit partial Boolean function depends on *k* bits and can be computed exactly by a 1-query quantum algorithm. That is, the function $F\left(f\right):=\mathrm{rank}\left({\left(\cdots ,P\left(x\right),\cdots \right)}_{f(x)=0}\right)+\mathrm{rank}\left({\left(\cdots ,P\left(x\right),\cdots \right)}_{f(x)=1}\right)-\left(2n+2-k\right)$ must be negative.

Compared with Theorem 1, Theorem 4 provides an approximate method on deciding the exact quantum query complexity of a partial Boolean function. Most importantly, the method in Theorem 4 is more efficient than Theorem 1. In order to construct the linear system in Theorem 1, we should observe at most |{a:f(a)=0}||{b:f(b)=1}| equations. In contrast, we only observe at most |{a:f(a)=0}|+|{b:f(b)=1}| inputs in Theorem 4. From this point, Theorem 4 is more efficient than Theorem 1. Note that the result in Theorem 4 is one-side exact. That is, if the exact quantum query complexity of a partial Boolean function *f* is bigger than 1 by Theorem 4, then the result is exact.

### 4.3. Estimating the Number of Partial Boolean Functions Depending on *k* Bits

In this subsection, let us evaluate the number $N_{1}\left(n,k\right)$ of *n*-bit partial Boolean functions which depend on *k* bits and can be computed exactly by a 1-query quantum algorithm.

As a preparation, the following lemma is necessary.

**Lemma** **4.** *Let the vector function $P\left(X_{k}\right)={\left(1,X_{k,1},X_{k,2},\cdots ,X_{k,n}\right)}^{T}$ for a string $X_{k}=X_{k,1}X_{k,2}\cdots X_{k,n}\in {\left\{0,1\right\}}^{n}$. If n≥2, for any j ∈{1,2,⋯,n+1} different linearly independent vectors $P(X_{1})$, $P(X_{2})$, ⋯,$P(X_{j})$, there exist at most $T_{j}\leq 2^{j-1}-j$ other different vectors $P(X_{j+1})$, $P(X_{j+2})$, ⋯,$P(X_{j+T_{j}})$ satisfying $\mathrm{rank}(\left[P\left(X_{1}\right),P\left(X_{2}\right),\cdots ,P\left(X_{j+T_{j}}\right)\right])=j$.*

By Theorem 4, the fifth result is the following.

**Theorem** **5.** *Let $N_{1}\left(n,k\right)$ be the number of all n-bit partial Boolean functions which depend on k bits and can be computed exactly by a 1-query quantum algorithm. If n≥3 and k≥2, then $N_{1}\left(n,k\right)\leq n^{2}2^{2^{n-1}\left(1+2^{2-k}\right)+2n^{2}}$.*

**Proof.**  Recall that all columns in the matrix ${\left(\cdots ,P\left(x\right),\cdots \right)}_{f(x)=b}$ traverse all legal inputs x∈D satisfying f(x)=b where the function vector $P\left(x\right)={\left(1,x_{1},x_{2},\cdots ,x_{n}\right)}^{T}$. Let $r_{0}$ denote the rank of ${\left(\cdots ,P\left(x\right),\cdots \right)}_{f(x)=0}$ and $r_{1}$ the rank of ${\left(\cdots ,P\left(x\right),\cdots \right)}_{f(x)=1}$. According to Theorem 4, $N_{1}\left(n,k\right)$ is not bigger than the number of all *n*-bit partial Boolean functions satisfying $r_{0}$$+r_{1}$≤2n+2−k and $1\leq r_{0},$$r_{1}\leq n$. For every fixed $\left(r_{0},r_{1}\right)$, an *n*-bit partial Boolean function can be determined using the following two steps.

In the first step, we choose $r_{0}$ linearly independent vectors

(27) $$P\left(X_{1}\right),P\left(X_{2}\right),\cdots ,P\left(X_{r_{0}}\right)$$

and $r_{1}$ linearly independent vectors

(28) $$P\left(Y_{1}\right),P\left(Y_{2}\right),\cdots ,P\left(Y_{r_{1}}\right)$$

for some $X_{1},X_{2},\cdots ,X_{r_{0}},Y_{1},Y_{2},\cdots ,Y_{r_{1}}\in {\left\{0,1\right\}}^{n}$, respectively. Here, $r_{0}$ linearly independent vectors $P(X_{1}),$$P(X_{2}),$⋯,$P(X_{r_{0}})$ correspond to $r_{0}$ different inputs with function value 0, and $r_{1}$ linearly independent vectors $P(Y_{1}),$$P(Y_{2}),$⋯,$P(Y_{r_{1}})$ correspond to $r_{1}$ different inputs with function value 1. For every fixed $\left(r_{0},r_{1}\right)$, the number of different selections (i.e., $\{P\left(X_{1}\right),$$P(X_{2}),$⋯,$P(X_{r_{0}})\}$ and $\{P\left(Y_{1}\right),$$P(Y_{2}),$⋯,$P(Y_{r_{1}})\}$) is not bigger than

(29) 2nr02n−r0r1≤2n(r0+r1)

for all n≥3 and k≥2.

In the second step, we add some other vectors $P(X_{r_{0}+1}),$⋯ and $P(Y_{r_{1}+1}),$⋯ to the sets $\{P\left(X_{1}\right),$$P(X_{2}),$⋯,$P(X_{r_{0}})\}$ and $\{P\left(Y_{1}\right),$$P(Y_{2}),$⋯,$P(Y_{r_{1}})\}$, respectively. Here, every newly added vector in the set $\{P\left(X_{r_{0}+1}\right),$⋯} should be represented linearly by the determined $r_{0}$ linearly independent vectors $P(X_{1}),$$P(X_{2}),$⋯,$P(X_{r_{0}})$ and every newly added vector in the set $\{P\left(Y_{r_{1}+1}\right),$⋯} should be represented linearly by the determined $r_{1}$ linearly independent vectors $P(Y_{1}),$$P(Y_{2}),$⋯,$P(Y_{r_{1}})$. After that, an *n*-bit partial Boolean function *f* is determined as follows. f(x)=0 for *x* in the set $\{X_{1},X_{2},$$\cdots ,X_{r_{0}},\cdots \}$, f(x)=1 for *x* in the set $\{Y_{1},Y_{2},$$\cdots ,Y_{r_{1}},\cdots \}$, and it is undefined for the rest cases. Using Equation (29) and Lemma 4, for every fixed $\left(r_{0},r_{1}\right)$, there are at most

(30) $$\begin{array}{c} 2^{n(r_{0}+r_{1})}2^{\left(2^{r_{0}-1}-r_{0}\right)}2^{\left(2^{r_{1}-1}-r_{1}\right)}<2^{2n^{2}}2^{\left(2^{n-1}+2^{n+1-k}\right)}=2^{2^{n-1}\left(1+2^{2-k}\right)+2n^{2}} \end{array}$$

partial Boolean functions with a pair of fixed $r_{0}$ and $r_{1}$. Note that the number of different $\left(r_{0},r_{1}\right)$ is not bigger than $n^{2}$. Thus, Theorem 5 has been proved. □

#### Discussions on Theorem 5

The most important contribution of Theorem 5 is to give an estimate on the number of partial Boolean functions with exact quantum 1-query complexity. This is the first non-trivial upper bound on this problem. In contrast, $3^{2^{n}}$ is the number of all *n*-bit partial Boolean functions, as each *n*-bit partial Boolean function corresponds to a string $f\left(0\right)f\left(1\right)\cdots f\left(2^{n}-1\right)\in {\left\{0,1,*\right\}}^{2^{n}}$. In fact, the exact quantum query complexity of any *n*-bit partial Boolean function is in the set {0,1,2,⋯,n}, which implies that $\max\left\{N_{j}\left(n,k\right):j,k\in \left\{0,1,2,\cdots ,n\right\}\right\}\geq \frac{3^{2^{n}}}{{\left(n+1\right)}^{2}}$. Here, the notation $N_{j}\left(n,k\right)$ is used to denote the number of *n*-bit partial Boolean functions which depend on *k* bits and can be computed exactly by a *j*-query quantum algorithm. Thus, all *n*-bit partial Boolean functions with exact quantum query complexity 1 only make up a very tiny proportion of all *n*-bit partial Boolean functions.

Furthermore, corresponding to Fact 1 in Ref. [23], the following Fact 2 is also applicable to all partial Boolean functions, as a common 1-query quantum algorithm computes the two partial Boolean functions.

Fact2. For any two partial Boolean functions *f* and *g* satisfying {x:g(x)=0}⊆{x:f(x)=0} and {y:g(y)=1}⊆{y:f(y)=1}, if *f* can be computed exactly by a 1-query quantum algorithm, then *g* can also be computed exactly by this 1-query quantum algorithm.

As we know, the partial Boolean function computed by Deutsch-Jozsa algorithm can be written as

(31) $$f\left(x\right)=\left\{\begin{array}{cc} 0, & \forall |x|=\frac{n}{2},\\ 1, & |x|=0,n. \end{array}\right.$$

By Fact 2, the exact quantum query complexity of any partial Boolean function *g* defined by

(32) $$g\left(x\right)=\left\{\begin{array}{cc} 0\;\mathrm{or}\;*, & \forall |x|=\frac{n}{2},\\ 1\;\mathrm{or}\;*, & |x|=0,n. \end{array}\right.$$

is also 1. The number of functions in this form is 3×(2nn2−1). Thus, for an even integer *n*, 3×(2nn2−1) is a trivial lower bound on the number of *n*-bit partial Boolean functions with exact quantum query complexity 1. In general, given an *n*-bit partial Boolean function with exact quantum query complexity 1, we can find out trivially at least $\left(2^{\left|\{a:f(a)=0\}\right|}-1\right)\times \left(2^{\left|\{b:f(b)=0\}\right|}-1\right)$ different partial Boolean functions with exact quantum query complexity 1. By Stirling’s approximation

(33) $$n!\approx \sqrt{2\pi n}{\left(\frac{n}{e}\right)}^{n},$$

we have

(34) nn2=n!n2!2≈2πn(ne)n2πn2n2en22=2πnπn2n.

Thus, the trivial lower bound

(35) 3×(2nn2−1)≈3×22πnπn2n.

Finally, the gap between the upper bound and the lower bound comes from the following two aspects. The first aspect is that the upper bound is obtained from a necessary condition (i.e., Theorem 4) and an approximate counting argument. The second aspect is that there exist many unknown partial Boolean functions with exact quantum query complexity 1.

## 5. Conclusions

Motivated by this issue of exact 1-query quantum model [9,10,15,22,23,24], in this paper, we have investigated the power and advantage of the exact 1-query quantum model for partial Boolean functions. Specifically, we have contributed two sufficient and necessary conditions for characterizing *n*-bit partial Boolean functions with exact quantum query complexity 1, and one necessary condition for characterizing *n*-bit partial Boolean functions that depend on *k*(k≤n) bits and can be computed exactly by a 1-query quantum algorithm. Using these characterizations, we have clarified all *n*-bit partial Boolean functions that depend on *n* bits and can be computed exactly by a 1-query quantum algorithm (in fact, n≤2 in this case, i.e. Theorem 2). Also, we have proved that the number of all *n*-bit partial Boolean functions with exact quantum query complexity 1 is quite small. As a result, the following two problems are worthy of further consideration.

- Find all (or some) non-trivial *n*-bit partial Boolean functions with exact quantum query complexity 1. This is an interesting problem for the following two aspects. On one hand, the upper bound (given by Theorem 5) of the actual number of partial Boolean functions in this class is quite big. On the other hand, known non-trivial *n*-bit partial Boolean functions in this class are still fairly rare [9,10,15,22,23,24].
- How many *n*-bit partial Boolean functions can be computed exactly (or with bounded-error) by *k*-query quantum algorithms for all k∈{2,3,⋯,n}? The solution of this problem is a quantitative evaluation of the advantage of the *k*-query quantum model. In contrast, the result of Ambainis et al. [14] is a qualitative evaluation of the advantage of the quantum query model.

## Data Availability

Not applicable.

## Appendix A. The Background of Definition 1

First, the following definition is used widely for total Boolean functions (i.e., $D={\left\{0,1\right\}}^{n}$) [14,28,31].

**Definition** **3.**
*[31]. Given an n-bit partial Boolean function $f:{\left\{0,1\right\}}^{n}\to \left\{0,1\right\}$, we say that f depends on the k-th (k∈{1,2,⋯,n}) bit if there exists a pair of inputs $X_{k},X_{k}^{\left\{k\right\}}\in {\left\{0,1\right\}}^{n}$ such that $1⊕f\left(X_{k}\right)=f\left(X_{k}^{\left\{k\right\}}\right)\in \left\{0,1\right\}$. Here, $X_{k}^{\left\{k\right\}}$ is the same as $X_{k}$ except for the k-th bit being flipped.*

Based on Definition 3, the statements “*f* depends on *k* bits” and “*f* depends on all bits (or *n* bits )” is also used widely for total Boolean functions (i.e., $D={\left\{0,1\right\}}^{n}$). Clearly, if a total Boolean function $f:{\left\{0,1\right\}}^{n}\to \left\{0,1\right\}$ depends on *k* bits, then we can always find out a sequence $i_{1}$, $i_{2}$, ⋯, $i_{k}$ such that *f* depends on the $i_{1}$-bit, the $i_{2}$-bit, ⋯, and the $i_{k}$-bit. Meanwhile, the unique multilinear polynomial representation of *f* is on the *k* variables (i.e., $x_{i_{1}}$, $x_{i_{2}}$, ⋯,$x_{i_{k}}$). However, for partial Boolean functions (i.e., $D\neq {\left\{0,1\right\}}^{n}$), things become different. On one hand, the even *n*-bit partial Boolean function *g* computed by Deutsch-Jozsa algorithm [10] does not depend on any bit (using Definition 3). On the other hand, the partial Boolean function *g* is not a constant function. Thus, Definition 3 is not proper for many partial Boolean functions, while the statements “*f* depends on *k* bits” and “*f* depends on all bits (or *n* bits )” should also be reconsidered. This point motivates us to use Definition 1 in this paper. Specifically, in the case of total Boolean functions, Definition 1 is consistent with Definition 3.

## Appendix B. Proof of Lemma 1

**Proof.** Recall that N(n)(n≥1) denotes the number of all *n*-bit partial Boolean functions depending on *n* bits. For any *k*-bit (k≥1) partial Boolean function f:D→{0,1} ($D\subseteq {\left\{0,1\right\}}^{k}$) depending on *k* bits (Note that *D* is not an empty set for k≥1), we construct at least $3^{2^{k}-1}$(k+1)-bit partial Boolean function *g* depending on k+1 bits by *f*. In fact, for any fixed $y=y_{1}y_{2}\cdots y_{k}\in D\subseteq {\left\{0,1\right\}}^{k}$, any (k+1)-bit partial Boolean function *g* defined by

(A1) $$\left\{\begin{array}{cc} g(x0)=f(x), & \forall x\in D,\\ g(x0)=*, & \forall x\in {\left\{0,1\right\}}^{k}\setminus D,\\ g(x1)=1⊕f(y), & x=y,\\ g(x1)\in \{0,1,*\}, & \forall x\in {\left\{0,1\right\}}^{k}\setminus \left\{y\right\} \end{array}\right.$$

is a (k+1)-bit partial Boolean function depending on (k+1) bits. For any k≥1, the last line in Equation (A1) implies that

(A2) $$\frac{N(k+1)}{N(k)}\geq 3^{2^{k}-1}.$$

Since N(1)=2 (i.e., (f(0),f(1))=(0,1) and (f(0),f(1))=(1,0)), we have

(A3) $$N\left(n\right)\geq 2\prod_{k=1}^{n-1}3^{2^{k}-1}=2\times 3^{2^{n}-n-1}.$$

The lemma has been proved. □

## Appendix C. Proof of Lemma 3

**Proof.** Assume that there exist at most k−1 non-zero vectors in all vectors $a_{Ø}$, $a_{\left\{1\right\}}$, $a_{\left\{2\right\}}$, ⋯,$a_{\left\{n\right\}}$. Then, there exist at least n−k+1 zero vectors $a_{\left\{i_{k}\right\}},$$a_{\left\{i_{k+1}\right\}},$⋯,$a_{\left\{i_{n}\right\}}$ where $i_{k}$, $i_{k+1}$, ⋯,$i_{n}$∈{1,2,⋯,n}. Note that all entries $a_{\left\{i_{r}\right\}}^{l}$ in $a_{\left\{i_{r}\right\}}$ are zeros for all l∈{1,2,⋯,p+q} and r∈{k,k+1,⋯,n}. Therefore,

(A4) $$\begin{array}{cc} f(x)= & |f_{1}{\left(x\right)|}^{2}+\cdots +{\left|f_{p}\left(x\right)\right|}^{2}\\ = & \sum_{l=1}^{p}{\left|a_{Ø}^{l}+\sum_{i\in \{1,2,\cdots ,n\}}a_{\left\{i\right\}}^{l}{\left(-1\right)}^{x_{i}}\right|}^{2}\\ = & \sum_{l=1}^{p}{\left|a_{Ø}^{l}+\sum_{r\in \{1,2,\cdots ,k-1\}}a_{\left\{i_{r}\right\}}^{l}{\left(-1\right)}^{x_{i_{r}}}\right|}^{2}. \end{array}$$

Obviously, the number of variables in this representation of *f* is at most k−1. Since *f* depends on *k* bits, this is a contradiction. Thus, the lemma has been proved. □

## Appendix D. Proof of Lemma 4

**Proof.** For every *k*≥j+1 and every fixed input $X_{r}=X_{r,1}X_{r,2}$⋯$X_{r,n}\in {\left\{0,1\right\}}^{n}$ where r∈{1,2,⋯,j,k}, let $P(X_{k})$=$s_{1}P\left(X_{1}\right)$ + $s_{2}P\left(X_{2}\right)$ + ⋯ + $s_{j}P\left(X_{j}\right)$ which is a linear system of equations on *j* undetermined variables $s_{1}$, $s_{2}$, ⋯,$s_{j}$. Note that this linear system of equations

(A5) $$\left\{\begin{array}{cc} \sum_{r=1}^{j}s_{r}=1, & \\ \sum_{r=1}^{j}s_{r}X_{r,i}=X_{k,i}, & i\in \{1,2,\cdots ,n\}. \end{array}\right.$$

consists of n+1 equations. Since vectors $P(X_{1})$, $P(X_{2})$, ⋯,$P(X_{j})$ is a base, the rank of the matrix ($P(X_{1})$, $P(X_{2})$, ⋯,$P(X_{j})$) is *j*. In other word, for all rows of the matrix ($P(X_{1})$, $P(X_{2})$, ⋯,$P(X_{j})$), we can find out a base which consists of *j* row vectors. After that, the augmented matrix of the linear system Equation (A5)

(A6) $$\left[\begin{array}{ccccc} 1 & 1 & \cdots & 1 & 1\\ X_{1,1} & X_{2,1} & \cdots & X_{j,1} & X_{k,1}\\ X_{1,2} & X_{2,2} & \cdots & X_{j,2} & X_{k,2}\\ \vdots & \vdots & \; & \vdots & \vdots \\ X_{1,n} & X_{2,n} & \cdots & X_{j,n} & X_{k,n} \end{array}\right]$$

can be transformed into

(A7) $$\left[\begin{array}{ccccc} 1 & 1 & \cdots & 1 & 1\\ X_{1,i_{1}} & X_{2,i_{1}} & \cdots & X_{j,i_{1}} & X_{k,i_{1}}\\ X_{1,i_{2}} & X_{2,i_{2}} & \cdots & X_{j,i_{2}} & X_{k,i_{2}}\\ \vdots & \vdots & \; & \vdots & \vdots \\ X_{1,i_{j-1}} & X_{2,i_{j-1}} & \cdots & X_{j,i_{j-1}} & X_{k,i_{j-1}}\\ 0 & 0 & \cdots & 0 & X_{k,i_{j}}^{\prime }\\ 0 & 0 & \cdots & 0 & X_{k,i_{j+1}}^{\prime }\\ \vdots & \vdots & \; & \vdots & \vdots \\ 0 & 0 & \cdots & 0 & X_{k,i_{n}}^{\prime } \end{array}\right]$$

where $\left\{i_{1},i_{2},\cdots ,i_{n}\right\}$={1,2,⋯,n}. According to the solution theory of linear system of equations, if any of $X_{k,i_{j}}^{\prime }$, $X_{k,i_{j+1}}^{\prime }$, ⋯, and $X_{k,i_{n}}^{\prime }$ is non-zero, then there exists no solution of the linear system Equation (A5) and the vector $P(X_{k})$ is not what we want. Otherwise, we can always get a solution

(A8) $$\left[\begin{array}{c} s_{1}\\ s_{2}\\ s_{3}\\ \vdots \\ s_{j} \end{array}\right]={\left[\begin{array}{cccc} 1 & 1 & \cdots & 1\\ X_{1,i_{1}} & X_{2,i_{1}} & \cdots & X_{j,i_{1}}\\ X_{1,i_{2}} & X_{2,i_{2}} & \cdots & X_{j,i_{2}}\\ \vdots & \vdots & \; & \vdots \\ X_{1,i_{j-1}} & X_{2,i_{j-1}} & \cdots & X_{j,i_{j-1}} \end{array}\right]}^{-1}\left[\begin{array}{c} 1\\ X_{k,i_{1}}\\ X_{k,i_{2}}\\ \vdots \\ X_{k,i_{j-1}} \end{array}\right].$$

As a result, for every $X_{k,i_{1}}X_{k,i_{2}}\cdots X_{k,i_{j-1}}\in {\left\{0,1\right\}}^{j-1}$, we either can get a unique string $X_{k}=$$X_{k,1}X_{k,2}\cdots X_{k,n}\in {\left\{0,1\right\}}^{n}$ satisfying $X_{k,i_{j}}^{\prime }=$$X_{k,i_{j+1}}^{\prime }$= ⋯ =$X_{k,i_{n}}^{\prime }=0$ in Equation (A7) or can not get a string $X_{r,1}X_{r,2}\cdots X_{r,n}\in {\left\{0,1\right\}}^{n}$ satisfying $X_{k,i_{j}}^{\prime }=$$X_{k,i_{j+1}}^{\prime }$= ⋯ =$X_{k,i_{n}}^{\prime }=0$ in Equation (A7). The lemma has been proved. □
